# Prenatal Glucocorticoid Exposure Modifies Endocrine Function and Behaviour for 3 Generations Following Maternal and Paternal Transmission

**DOI:** 10.1038/s41598-017-11635-w

**Published:** 2017-09-18

**Authors:** Vasilis G. Moisiadis, Andrea Constantinof, Alisa Kostaki, Moshe Szyf, Stephen G. Matthews

**Affiliations:** 1 Department of Physiology, University of Toronto, Toronto, ON M5S1A8 Canada; 2 Department of Pharmacology & Therapeutics, Sackler Program for Epigenetics & Psychobiology, McGill University, Montreal, QC H3G1Y6 Canada; 3 Department of Obstetrics and Gynecology, Toronto, ON M5S1A8 Canada; 4 Department of Medicine, University of Toronto, Toronto, ON M5S1A8 Canada

## Abstract

Fetal exposure to high levels of glucocorticoids programs long-term changes in the physiologic stress response and behaviours. However, it is not known whether effects manifest in subsequent generations of offspring following maternal (MT) or paternal (PT) transmission. We treated pregnant guinea pigs with three courses of saline or synthetic glucocorticoid (sGC) at a clinically relevant dose. Altered cortisol response to stress and behaviours transmitted to juvenile female and male F_2_ and F_3_ offspring from both parental lines. Behavioural effects of sGC in F_1_-F_3_ PT females associated with altered expression of genes in the prefrontal cortex and hypothalamic paraventricular nucleus (PVN). Exposure to sGC programmed large transgenerational changes in PVN gene expression, including type II diabetes, thermoregulation, and collagen formation gene networks. We demonstrate transgenerational programming to F_3_ following antenatal sGC. Transmission is sex- and generation-dependent, occurring through both parental lines. Paternal transmission to F_3_ females strongly implicates epigenetic mechanisms of transmission.

## Introduction

Glucocorticoids (GC) are a potent developmental trigger during pregnancy. The late-gestation GC surge promotes rapid maturation of fetal organs, including the lungs and brain^1^. However, fetal exposure to high levels of GC prior to the surge, either through maternal stress or treatment with synthetic glucocorticoids (sGC), alters the trajectory of development and programs stress-associated behaviours (e.g. locomotor activity, attention, anxiety) through altered gene expression in the medial prefrontal cortex (mPFC) and hypothalamic-pituitary-adrenal (HPA) axis^2–5^.

Women at risk for preterm birth between 24–34 weeks gestation receive sGC to improve infant respiratory outcomes^6^. While a single course of sGC is currently the gold-standard therapy, during the 1990’s and early 2000’s multiple course treatment with sGC was common^7,8^. Antenatal exposure to multiple courses of sGC has been associated with hyperactivity^9^, impaired attention^10,11^, and neurodevelopmental impairment^12^ in young children and animals^2^. It is imperative that the long-term effects of antenatal exposure to multiple courses of sGC continue to be investigated since the use of a ‘rescue’ (i.e. a second) course of sGC has recently re-introduced the practice of multiple course administration^13,14^.

While emerging evidence indicates that antenatal exposure to sGC modifies cardiometabolic and endocrine phenotypes across two generations^15–19^, the effects on HPA activity and stress-related behaviours past the second generation (F_2_) are not known. Furthermore, only a few studies have investigated programming by antenatal sGC via the paternal line^19,20^ though there is strong evidence for paternal transmission to F_2_ and F_3_ following antenatal exposure to stressors^21–24^. We investigated whether antenatal exposure to sGC in the guinea pig can affect HPA activity and stress-related behaviours in juvenile offspring across multiple generations. The guinea pig was chosen as a model since they share similar patterns of brain development and placentation with the human, and the long gestation, approximately 69 days, allows for targeting specific phases of development^25,26^. Additionally, the primary GC in guinea pigs is cortisol (as in humans), as compared to corticosterone in mice and rats. We focused our investigation on juvenile offspring since previous studies have indicated that prenatal exposure to sGC results in particularly strong neuroendocrine and behavioural effects in children and young animals. We hypothesized that prenatal sGC exposure results in modified: 1) HPA responsiveness to stress; 2) locomotor activity, anxiety-like behaviour, and sensorimotor gating (attention); 3) altered transcription of key regulatory genes in the mPFC; and 4) altered patterns of gene expression in the PVN. These effects would manifest across three generations, be sex-specific, and depend on the route of transmission (maternal vs. paternal). We show that; 1) transgenerational inheritance in mammals via the paternal lineage does occur, and 2) the effects of a clinically relevant dose of antenatal sGC can be transmitted over multiple generations. These findings have major implications for the study of developmental health and disease.

## Results

### Reproduction

**Figure 1:**
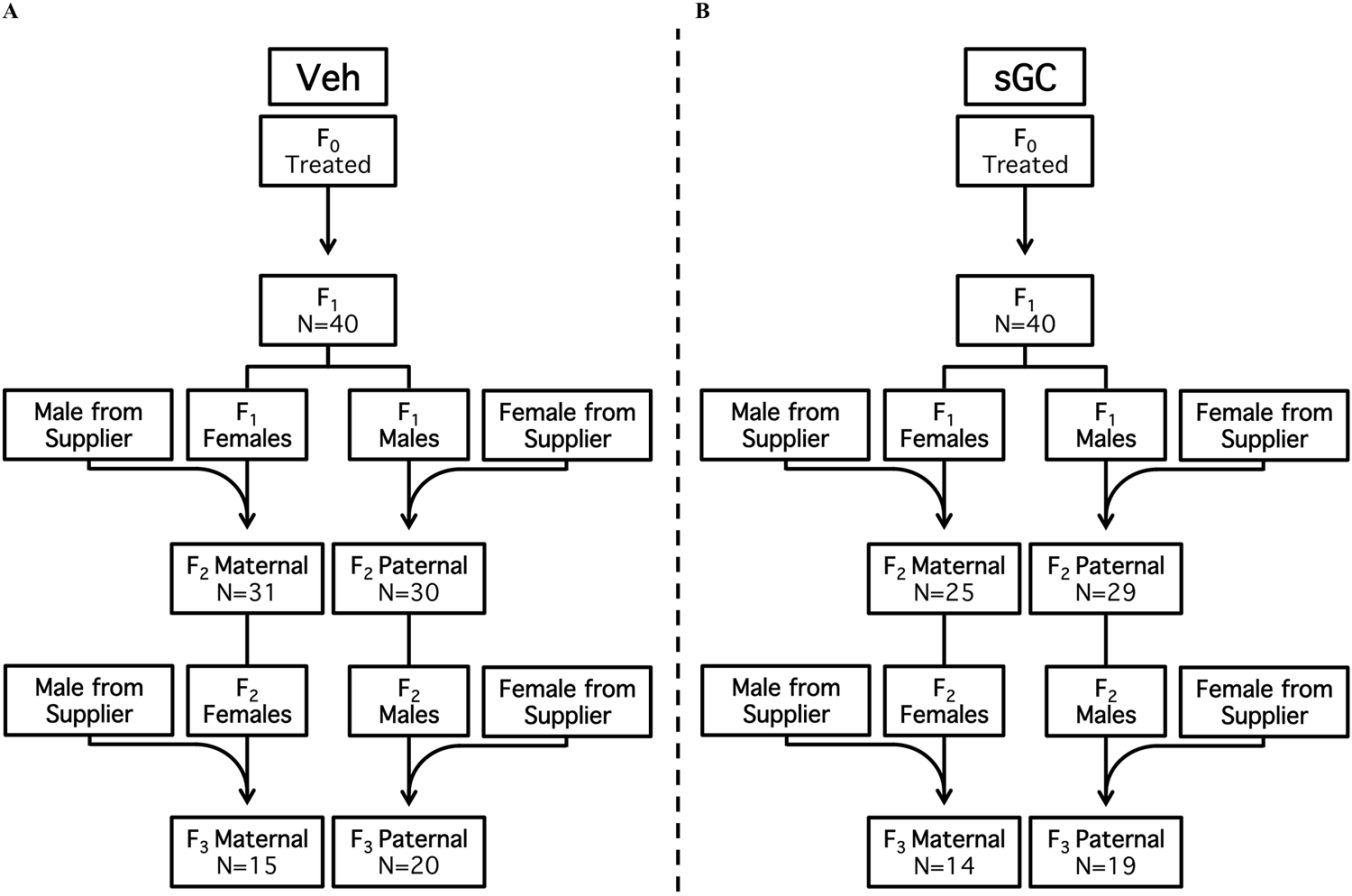
Breeding scheme for (**A**) Veh and (**B**) sGC groups. Maternal (Mat) and paternal (Pat) lines originate from F_1_ female and male breeders (respectively). N represents the total number of independent litters for each treatment, breeding line and generation.

There was no significant effect of sGC treatment on breeding parameters (age at conception-females 110.0 d ± 1.44; males 137.6 d ± 4.5, number of breeding attempts, length of gestation, pregnancy weight gain, litter size and sex ratio) in the F_0-_F_2_ pregnancies (data not shown). There was a trend towards exposure to sGC (F_0_) increasing the length of gestation for the F_2_ paternal transmission (PT) pregnancy compared to Veh (Veh 68.5 d ± 0.40, sGC 69.6 d ± 0.35; t_23_ = 2.003; P = 0.053). The breeding paradigm is presented in Fig. 1.

### Growth

#### F_1_ & F_2_

There was no significant effect of sGC treatment on body measures (body length, abdominal circumference, head length and circumference), birth weight, or weight gain in F_1_ or F_2_ offspring for either transmission route (data not shown).

#### F_3_

Exposure to sGC (F_0_) increased abdominal circumference compared to Veh in PT males on PND 0 and 20 (PND 0: Veh 8.06 cm ± 0.18, sGC 8.23 cm ± 0.27; PND20: Veh 10.75 cm ± 0.25, sGC 11.33 cm ± 0.22; F_(1, 23)_ = 5.012; P < 0.05). Exposure to sGC also reduced adrenal:body-weight ratio for PT females (Veh 2.00e-4 ± 7.41e-6, sGC 1.73e-4 ± 5.85e-6; t_16_ = 2.804; P < 0.05). There were no other effects of sGC treatment on growth for either transmission route (data not shown).

### HPA Function

**Figure 2:**
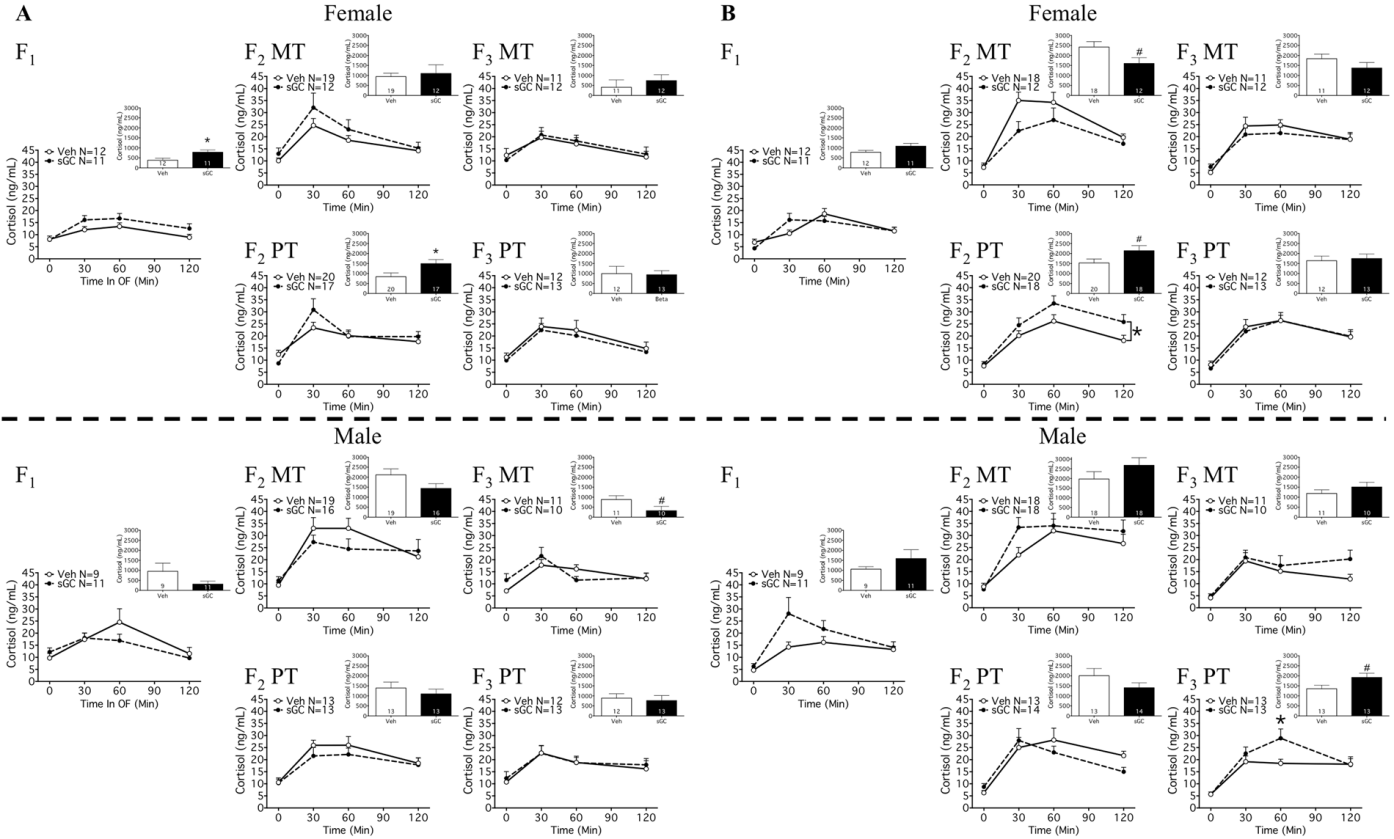
HPA response to open field stress on (**A**) PND 19 and (**B**) PND 24 for F_1_-F_3_ female and male MT (Maternal Transmission) and PT (Paternal Transmission) offspring. Veh (Vehicle) open symbols, sGC (synthetic glucocorticoid) closed symbols. Insert: salivary cortisol AUC_N_ (net area under the curve). Data are expressed as mean ± SEM. A significant difference between Veh and sGC groups is represented as follows: *P < 0.05; ^#^P < 0.075.

There was an effect of time as a repeated measure at all ages for all groups (Fig. 2A and B; P < 0.05).

#### F_1_

Antenatal treatment with sGC resulted in an increased cortisol response to open field (OF) stress compared with Veh for females on PND 19 (Fig. 2A; AUC_N_; t_20_ = 2.596; P < 0.05). There were no effects of treatment with sGC on HPA response to stress for females on PND 24 or for males (Fig. 2A and B).

#### F_2_

Exposure to sGC (F_0_) resulted in an increased cortisol response for PT females on PND 19 (Fig. 2A; AUC_N_; t_35_ = 2.348; P < 0.05), and a greater total cortisol response to stress for PT females on PND 24 (Fig. 2B; AUC_T_: Veh 2441.0 ng/mL ± 194.3, sGC 3143.0 ng/mL ± 247.1; t_36_ = 2.255; P < 0.05). There was a trend towards exposure to sGC (F_0_) reducing the cortisol response to stress compared to Veh for maternal transmission (MT) females on PND 24 (Fig. 2B; AUC_N_; t_28_ = 2.021; P = 0.053). There were no effects of sGC exposure on HPA response to stress for F_2_ MT and PT males (Fig. 2A and B).

#### F_3_

There was an interaction between treatment and time on the cortisol response to stress for PT males on PND 24 (Fig. 2B; F_(3, 72)_ = 3.383; P < 0.05), and post-hoc analysis revealed increased cortisol at 60 min compared to Veh (Veh 18.48 ng/mL ± 1.74, sGC 28.87 ng/mL ± 3.86; Tukey HSD; P < 0.05). There were trends towards exposure to sGC reducing cortisol response to stress compared to Veh for MT males on PND 19 (Fig. 2A; t_19_ = 1.906; P = 0.072) and increasing cortisol response to stress for PT males on PND 24 (Fig. 2B: AUC_N_; t_24_ = 2.051; P = 0.051). There were no effects of sGC exposure on HPA response to stress for MT or PT females (Fig. 2A and B).

### Open Field Behaviour

#### F_1_

**Figure 3:**
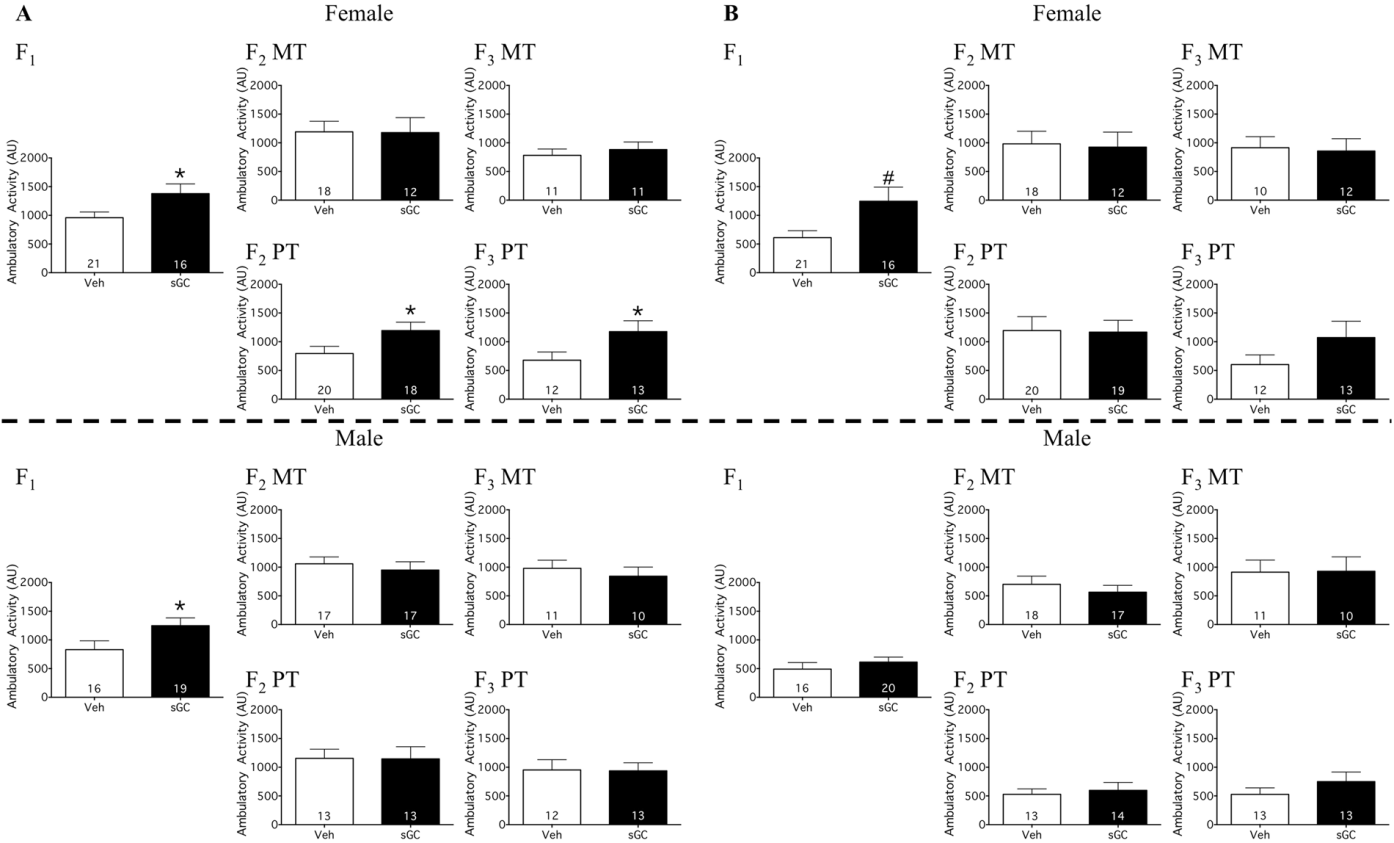
Total locomotor activity in the open field on (**A**) PND 19 and (**B**) PND 24 for F_1_-F_3_ female and male MT (Maternal Transmission) and PT (Paternal Transmission) offspring. Veh (Vehicle) open bars, sGC (synthetic glucocorticoid) closed bars. Data are expressed as mean ± SEM. A significant difference between Veh and sGC groups is represented as follows: *P < 0.05; ^#^P < 0.075.

Antenatal treatment with sGC resulted in increased total activity in the OF compared to Veh for females (Fig. 3A; t_35_ = 2.251; P < 0.05) and males on PND 19 (Fig. 3A; t_33_ = 2.039; P < 0.05). There was a trend towards sGC treatment increasing total activity compared to Veh for females on PND 24 (Fig. 3B; t_35_ = 1.958; P = 0.058). There were also trends towards treatment with sGC increasing the distance traveled compared to Veh for males on PND 19 (Veh 833.2 cm ± 158.7, sGC 1233.0 cm ± 143.2; t_33_ = 1.875; P = 0.070) and females on PND 24 (Veh 582.9 cm ± 117.4, sGC 1213.0 cm ± 243.9; t_33_ = 1.947; P = 0.058). There were no effects of sGC treatment on total locomotor activity for males on PND 24 (Fig. 3A and B), distance traveled by females on PND 19 and males on PND 24 (data not shown), or on the percent of time that F_1_ offspring spent at the perimeter of the OF chamber (data not shown).

#### F_2_

For PT females, exposure to sGC (F_0_) resulted in increased total activity (Fig. 3A; t_36_ = 2.092; P < 0.05), increased distance traveled (Veh 797.3 cm ± 130.0, sGC 1196.0 cm ± 146.6; t_36_ = 2.042; P < 0.05), and reduced the percent time spent around the perimeter compared to Veh on PND 19 (Veh 96.9% ± 0.92, sGC 88.3% ± 1.66; U = 38; P < 0.05). There were no effects of exposure to sGC on total locomotor activity (Fig. 3A and B), distance traveled (data not shown), or the percent time spent around the perimeter (data not shown) for MT offspring, PT females on PND 24, or PT males.

#### F_3_

Exposure to sGC (F_0_) resulted in increased total locomotor activity compared to Veh for PT females on PND 19 (Fig. 3A; t_23_ = 2.065; P < 0.05). There was a trend towards sGC increasing the distance traveled compared to Veh for PT females on PND 19 (Veh 668.0 cm ± 145.2, sGC 1173.0 cm ± 196.3; t_23_ = 2.038; P = 0.053). Exposure to sGC also reduced the percent of time around the perimeter compared to Veh for MT males on PND 19 (Veh 91.84% ± 3.14, sGC 57.0% ± 10.21; U = 22; P < 0.05). There was a trend towards sGC reducing the percent of time around the perimeter compared to Veh for PT females on PND 24 (Veh 96.71% ± 1.51, sGC 92.55% ± 1.38; t_23_ = 2.036; P = 0.053). There were no effects of exposure to sGC on total locomotor activity (Fig. 3A and B) or distance traveled (data not shown) for MT offspring, PT males, or PT females on PND 24. There were also no effects of exposure to sGC on the percent of time around the perimeter (data not shown) for MT females, MT males on PND 24, PT females on PND 19, or PT males.

### Acoustic Startle Response and Prepulse Inhibition

**Figure 4:**
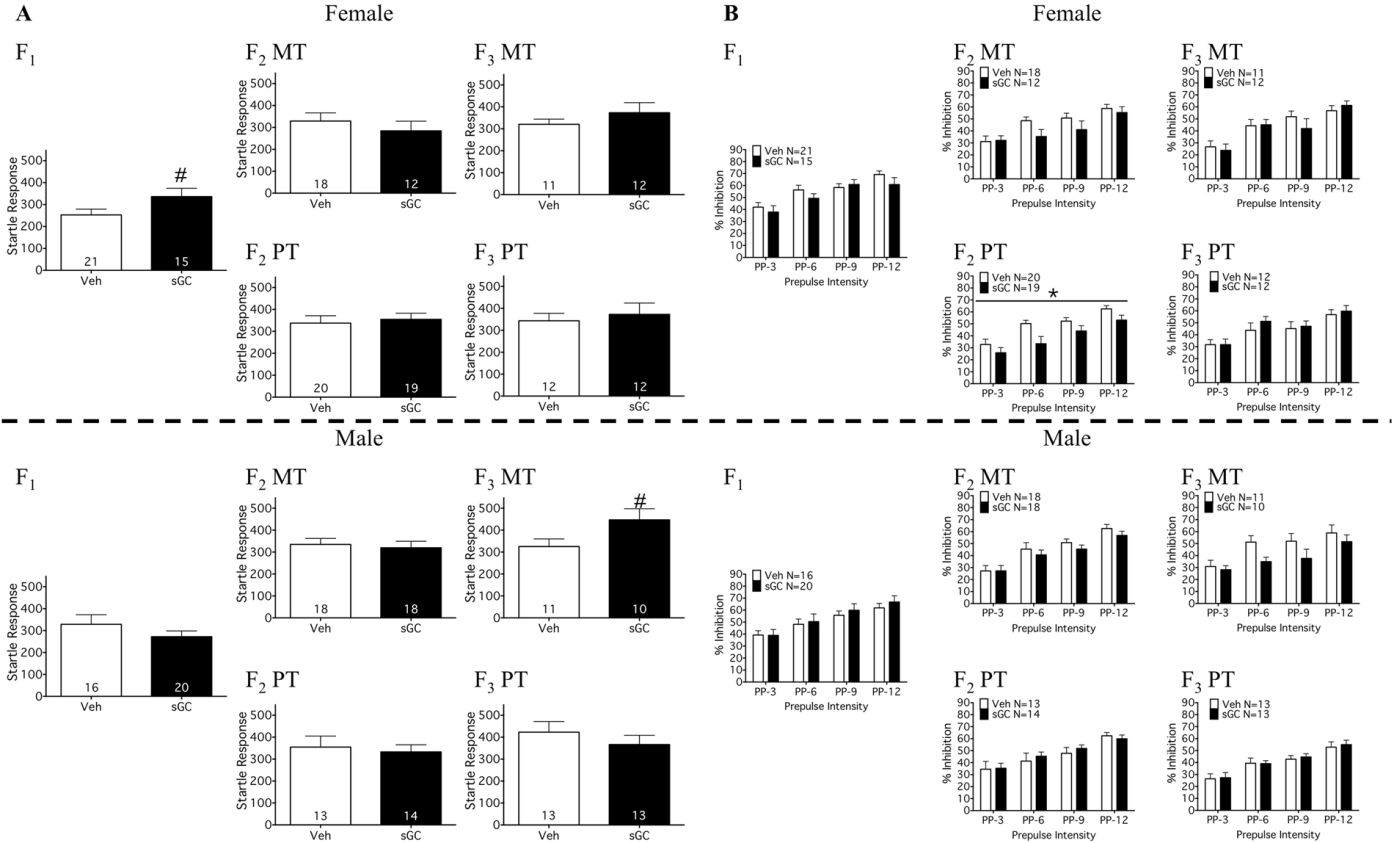
(**A**) Acoustic startle response (ASR; PND 23) and (**B**) percent inhibition of the startle response at increasing prepulse intensities (PPI; PND 23) for F_1_-F_3_ female and male MT (Maternal Transmission) and PT (Paternal Transmission) offspring. Veh (Vehicle) open bars, sGC (synthetic glucocorticoid) closed bars. Data are expressed as mean ± SEM. A significant difference between Veh and sGC groups is represented as follows: *P < 0.05; ^#^P < 0.075.

There was an effect of prepulse as a repeated measure at all prepulse intensities in all groups (Fig. 4B; P < 0.05), indicating that percent inhibition increased as a function of prepulse intensity.

#### F_1_

There was no significant effect of sGC treatment on the ASR or PPI for F_1_ offspring (Fig. 4A and B), but there was a trend towards increased ASR compared to Veh for females (Fig. 4A; t_34_ = 1.864; P = 0.071).

#### F_3_ & F_3_

There were no effects of exposure to sGC on the ASR in F_2_ or F_3_ (Fig. 4A), though there was a trend towards increased ASR compared to Veh for F_3_ MT males (Fig. 4A; t_19_ = 2.003; P = 0.06). Exposure to sGC resulted in reduced PPI compared to Veh for F_2_ PT females (Fig. 4B; F_(1, 37)_ = 5.08; P < 0.05). There were no effects of exposure to sGC on PPI for F_2_ or F_3_ MT offspring, F_2_ PT males, or F_3_ PT offspring (Fig. 4B).

### Locomotor Activity: Home Cage

**Figure 5:**
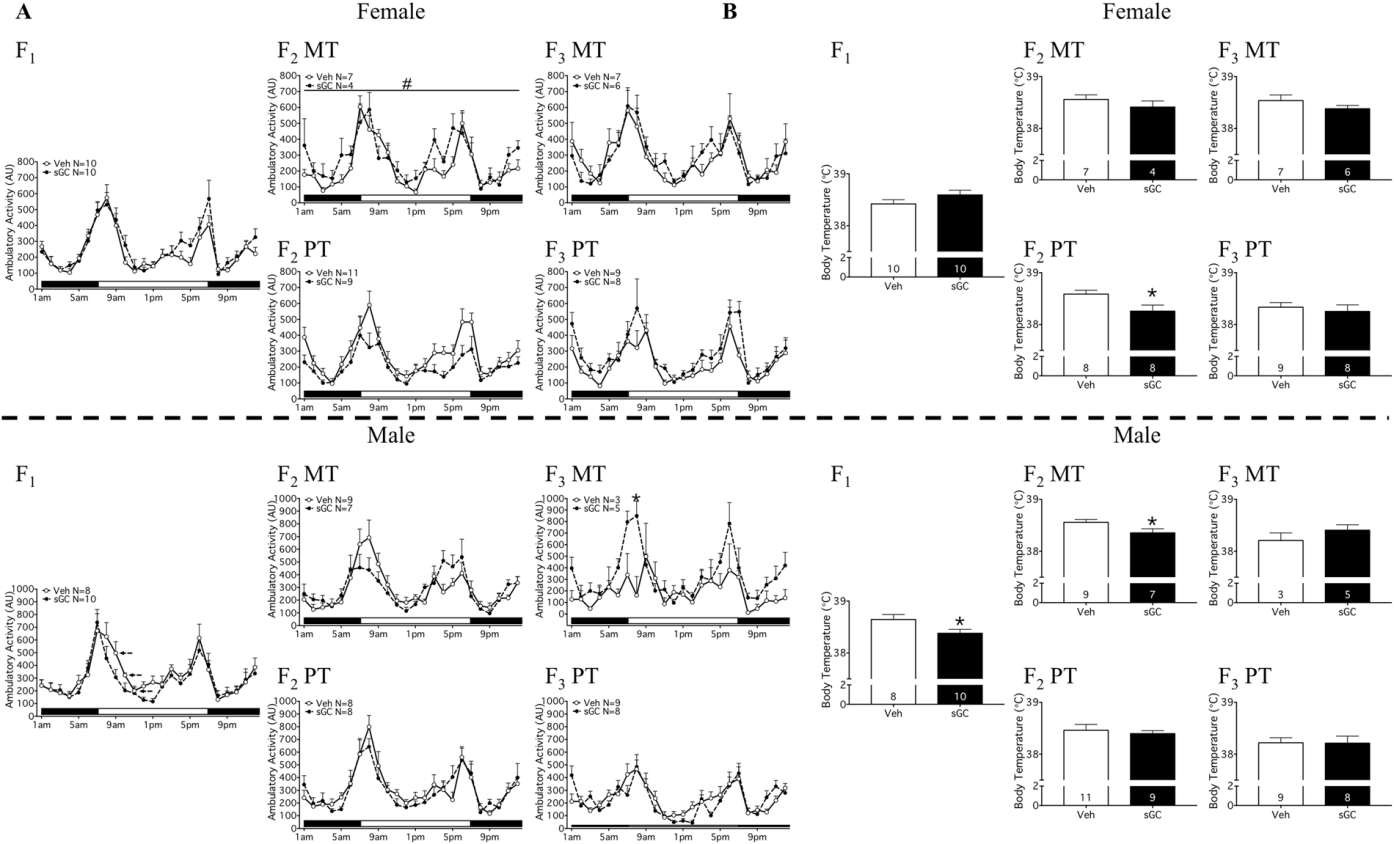
(**A**) Locomotor activity and (**B**) average body temperature over 24-hour period in the animal’s home cage for F_1_-F_3_ female and male MT (Maternal Transmission) and PT (Paternal Transmission) offspring. Veh (Vehicle) open symbols/bars, sGC (synthetic glucocorticoid) closed symbols/bars. (**A**) X-axis: solid bar indicates lights off, open bar indicates lights on. Data are expressed as mean ± SEM. A significant difference between Veh and sGC groups is represented as follows: *P < 0.05; ^#^P = 0.068. A significant 1-hour phase difference between Veh and Beta groups is represented as follows: P < 0.05.

There was an effect of time of day on locomotor activity in all groups (Fig. 5A; P < 0.05).

#### F_1_

In males, there was a within treatment effect of time (F_(23, 368)_ = 1.97; P < 0.05), and post-hoc analysis revealed that treatment with sGC resulted in a 1-hour phase advance in the profile of activity compared to Veh for males from 0900 h–1100 h (Fig. 5A; Tukey HSD; P < 0.05 at each time point). There was no effect of sGC treatment on locomotor activity for females (Fig. 5A).

#### F_2_

There was no significant effect of exposure to sGC (F_0_) on locomotor activity for either transmission route (Fig. 5A). There was a trend towards exposure to sGC increasing activity compared to Veh for MT females (Fig. 5A; F_(1, 9)_ = 4.304; P = 0.068).

#### F_3_

There was a significant interaction between treatment and time of day on locomotor activity for MT males (Fig. 5A; F_(23, 138)_ = 2.136; P < 0.05), and post-hoc analysis revealed that exposure to sGC increased locomotor activity at 0800 h compared to Veh (Fig. 5A; Tukey HSD; P < 0.05). There were no other effects of exposure to sGC on locomotor activity for either transmission route (Fig. 5A).

### Body Temperature

#### F_1_

sGC treatment reduced overall body temperature compared to Veh for males (Fig. 5B; t_16_ = 2.215; P < 0.05). There was no effect of sGC treatment on overall body temperature for females or on rhythmicity in males or females (Fig. 5B).

#### F_2_ & F_3_

Overall body temperature was reduced by exposure to sGC in F_2_ MT males (Fig. 5B; t_14_ = 2.173; P < 0.05) and F_2_ PT females compared to Veh (Fig. 5B; t_18_ = 2.449; P < 0.05). There were no other effects of exposure to sGC on overall body temperature or rhythmicity in F_2_ of in F_3_ (Fig. 5B).

### Medial Prefrontal Cortex: Gene Expression

#### F_1_

**Figure 6:**
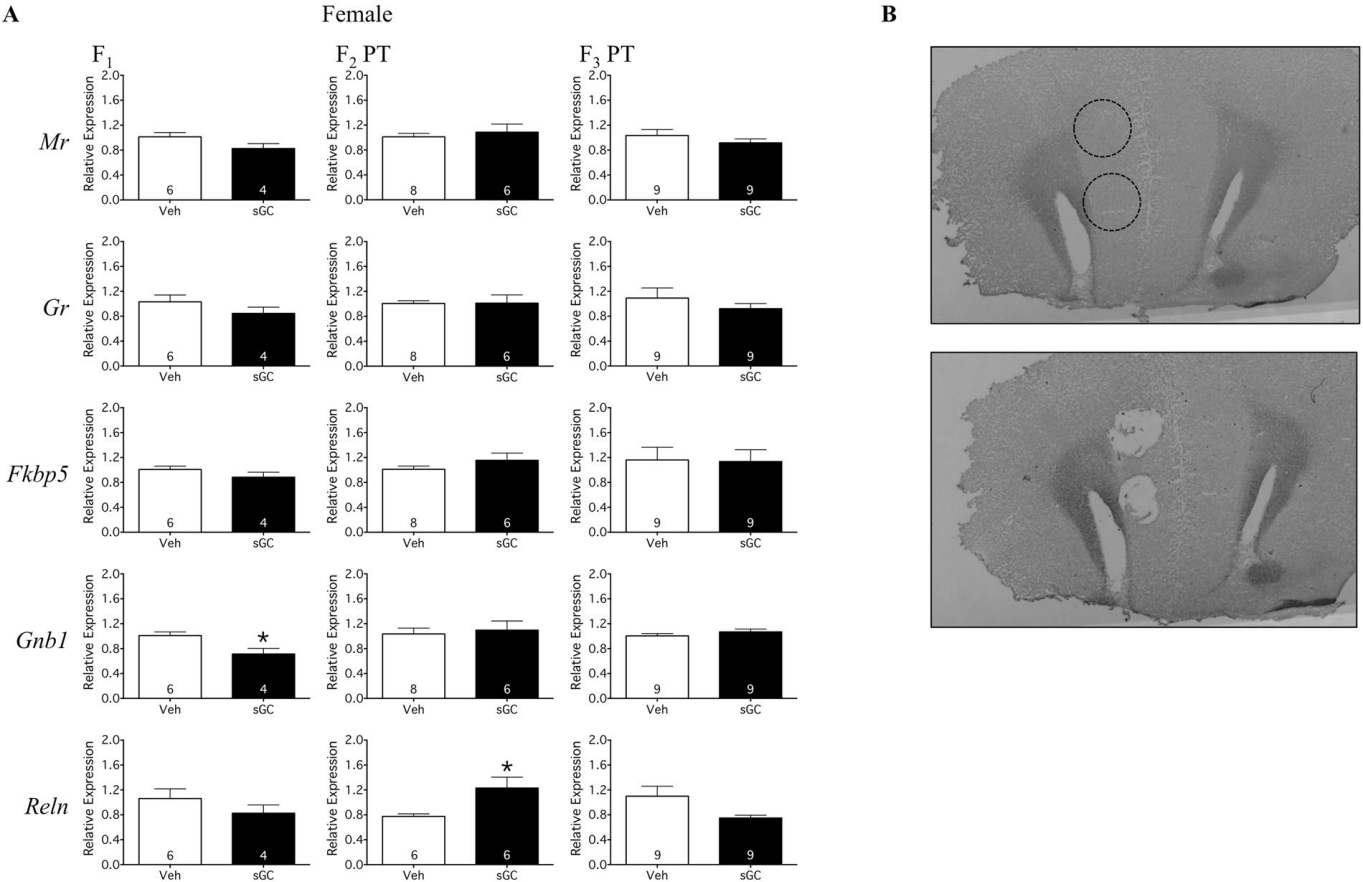
(**A**) Gene expression in the mPFC relative to the housekeeping gene *Gapdh* for F_1_-F_3_ female PT (Paternal Transmission) offspring. Veh (Vehicle) open bars, sGC (synthetic glucocorticoid) closed bars. Data are expressed as mean ± SEM. A significant difference between Veh and sGC groups is represented as follows: *P < 0.05. (**B**) Representative sections of the mPFC before and after tissue punch. Dashed line indicates site of punch.

Antenatal treatment with sGC reduced the expression of *Gnb1* mRNA in the F_1_ female mPFC compared to Veh (Fig. 6A; t_8_ = 2.885; P < 0.05). There was no effect of sGC treatment on the expression of *Mr*, *Gr*, *Fkbp5*, or *Reln* mRNA (Fig. 6A).

#### F_2_ & F_3_

In F_2_, exposure to sGC resulted in increased expression of *Reln* mRNA in the PT female mPFC compared to Veh (Fig. 6A; t_10_ = 2.839; P < 0.05). There was no effect of sGC treatment on the expression of *Mr*, *Gr, Fkbp5*, or *Gnb1* mRNA in F_2_, or on the expression of any gene in the F_3_ mPFC (Fig. 6A).

### Hypothalamic Paraventricular Nucleus: Gene Expression

#### F_1_

**Figure 7:**
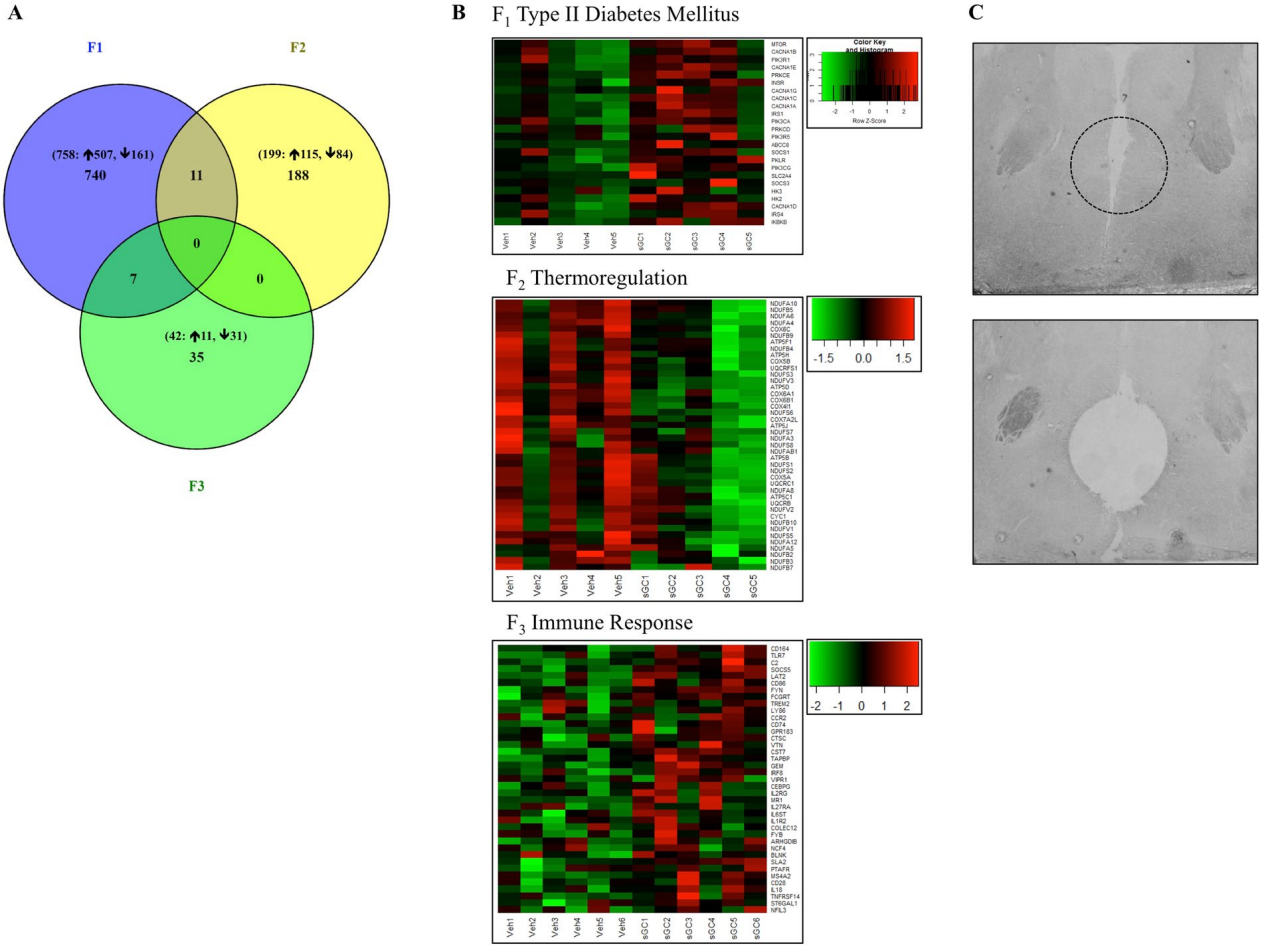
(**A**) Venn diagram illustrating the number of genes that are significantly differentially expressed in the PVN from F_1_-F_3_ female PT (Paternal Transmission) and the number of genes that overlap between generations. Numbers in brackets indicate the total number of differentially expressed genes and the number of genes with increased or decreased expression in the sGC group. (**B**) Heat maps of significantly enriched gene sets in the PVN for F_1_-F_3_ PT female offspring (NES > 1.6, FDR < 0.25). Each row represents one gene, each column represents one animal. Green represents lowly expressed genes and red represents highly expressed genes. (**C**) Representative images of the PVN before and after tissue punch. Dashed line indicates site of punch.

758 genes were differentially expressed following antenatal treatment with sGC in F_1_ females compared to Veh (Fig. 7A; FDR < 0.05); expression was increased in 597 genes and reduced in 161. Gene set enrichment analysis (GSEA) for differential gene expression between the Veh and sGC groups revealed enrichment of 160 gene sets (Supplementary Table 1; NES > 1.6, FDR < 0.25); 72 gene sets were positively enriched (i.e. increased expression with sGC treatment) and 88 were negatively enriched (i.e. decreased expression). 14 gene sets were associated with known behavioural, metabolic, and molecular effects of sGC treatment (Table 1; NES > 1.6, FDR < 0.25), including type II diabetes mellitus (Fig. 7B; NES > 1.6, FDR < 0.25). A custom HPA Activity gene set (Table 3) was not enriched (Table 1; NES = 0.92, FDR = 0.618), although the expression of *Nr3c2* (encoding *Mr)* was significantly increased; no other classic HPA genes were differentially expressed.

**Table 1:** PVN Gene Sets Associated with Phenotypes.

Phenotype	Gene Set	Type	Generation	Effect of sGC	Size	NES	P-Value	FDR
HPA Activity	STRESS_ASSOCIATED_GENES	Custom	F_1_	Positive	18	0.92	0.618	0.618
			F_2_	Positive	18	1.25	0.154	0.154
			F_3_	Negative	18	1.06	0.409	0.409
One-Carbon Metabolism and Methylation	S_ADENOSYLMETHIONINE_DEPENDENT_METHYLTRANSFERASE_ACTIVITY	c5	F_1_	Negative	18	1.60	0.019	0.119
Circadian Rhythm	REACTOME_RORA_ACTIVATES_CIRCADIAN_EXPRESSION	c2	F_1_	Positive	21	1.80	<0.001	0.017
	REACTOME_CIRCADIAN_REPRESSION_OF_EXPRESSION_BY_REV_ERBA	c2		Positive	19	1.79	0.001	0.018
Metabolic Dysfunction	KEGG_TYPE_II_DIABETES_MELLITUS	c2	F_1_	Positive	36	1.71	0.001	0.031
	FEEDING_BEHAVIOR	c5	F_2_	Positive	16	1.74	0.016	0.124
	DIGESTION	c5	F_3_	Negative	17	1.75	0.003	0.571
Thermoregulation	REACTOME_RESPIRATORY_ELECTRON_TRANSPORT_ATP_SYNTHESIS_BY_CHEMIOSMOTIC_COUPLING_AND_HEAT_PRODUCTION_BY_UNCOUPLING_PROTEINS_	c2	F_1_	Negative	54	3.11	<0.001	<0.001
			F_2_	Negative	55	1.86	<0.001	0.021
Collagen and Matrix	REACTOME_COLLAGEN_FORMATION	c2	F_1_	Positive	34	1.87	<0.001	0.006
			F_3_	Positive	47	1.62	0.017	0.174
	EXTRACELLULAR_MATRIX_STRUCTURAL_CONSTITUENT	c5	F_1_	Positive	19	1.92	<0.001	0.005
	REACTOME_EXTRACELLULAR_MATRIX_ORGANIZATION	c2	F_1_	Positive	44	1.78	0.001	0.017
Immune Function	IMMUNE_RESPONSE	c5	F_3_	Positive	107	1.89	<0.001	0.162
	CELLULAR_DEFENSE_RESPONSE	c5	F_3_	Positive	21	1.87	0.002	0.116
	KEGG_CYTOKINE_CYTOKINE_RECEPTOR_INTERACTION	c2	F_1_	Positive	100	1.77	<0.001	0.028
			F_2_	Negative	103	1.66	<0.001	0.070
	REACTOME_CELL_SURFACE_INTERACTIONS_AT_THE_VASCULAR_WALL	c2	F_2_	Negative	54	1.62	0.005	0.125
			F_3_	Positive	57	1.89	<0.001	0.017
Signaling	REACTOME_PEPTIDE_LIGAND_BINDING_RECEPTORS	c2	F_2_	Positive	77	1.85	<0.001	0.106
			F_3_	Negative	79	2.07	<0.001	0.001

Gene sets that are enriched in the F_1_-F_3_ female PT PVN and associate with offspring phenotypes. Type: collections of gene sets; c5 = gene ontology, c2 = curated gene sets from published data sets. Effect of sGC: describes relationship between treatment with sGC and the expression of genes within a gene set, positive-increased expression, negative-decreased expression. Size: number of genes in the gene set. NES: normalized enrichment score used to compare gene sets of different sizes. FDR: false discovery rate, the probability that a gene set with a specific NES is a false positive.

**Table 3:** Custom HPA Activity Gene Set: STRESS_ASSOCIATED_GENES.

Gene	F_1_			F_2_			F_3_		
	Log_2_ Fold Change	P-Value	FDR	Log_2_ Fold Change	P-Value	FDR	Log_2_ Fold Change	P-Value	FDR
*Arntl*	−0.089	0.326	0.603	−0.027	0.788	0.999	0.103	0.056	0.636
*Avp*	−1.016	0.026	0.161	0.541	0.043	0.508	−0.110	0.673	0.985
*Bdnf*	−0.082	0.607	0.812	0.130	0.431	0.999	−0.215	0.111	0.768
*Clock*	0.405	0.000	0.003	0.092	0.344	0.999	−0.023	0.667	0.984
*Crh*	−0.991	0.004	0.057	0.675	0.003	0.118	−0.075	0.719	0.991
*Crhr2*	0.322	0.028	0.168	−0.026	0.852	0.999	−0.039	0.481	0.967
*Cry1*	0.045	0.671	0.849	0.011	0.924	0.999	−0.037	0.624	0.979
*Cry2*	−0.019	0.843	0.934	0.013	0.898	0.999	−0.051	0.288	0.910
*Fkbp5*	0.433	0.071	0.276	0.115	0.555	0.999	0.081	0.501	0.971
*Gad1*	0.362	0.058	0.247	−0.076	0.755	0.999	−0.094	0.362	0.937
*Hsd11b2*	−0.302	0.287	0.566	−0.287	0.241	0.982	−0.105	0.631	0.979
*Jun*	0.024	0.810	0.918	0.172	0.111	0.773	−0.030	0.605	0.978
*Nfkb1*	0.177	0.178	0.445	0.040	0.767	0.999	−0.157	0.084	0.711
*Nr3c1*	0.070	0.518	0.756	0.059	0.550	0.999	0.093	0.024	0.490
*Nr3c2*	0.326	0.001	0.026	−0.088	0.432	0.999	−0.062	0.318	0.920
*Per1*	0.131	0.262	0.543	0.036	0.726	0.999	−0.010	0.901	0.996
*Per2*	0.293	0.025	0.156	0.059	0.622	0.999	0.013	0.884	0.996
*Trh*	−0.823	0.002	0.041	0.264	0.163	0.883	−0.139	0.602	0.978

Expression data for individual genes in the HPA activity gene set, STRESS_ASSOCIATED_GENES, in the F_1_-F_3_ female PT PVN. Log_2_ fold-change from Veh. Unadjusted P-value. FDR adjusted.

#### F_2_ & F_3_

Exposure (F_0_) to sGC resulted in the differential expression of 199 genes in F_2_ (115 increased, 84 reduced) and 42 genes in F_3_ (11 increased, 31 reduced; Fig. 7A; FDR < 0.05). 11 genes were differentially expressed in F_1_ and F_2_ (*Myo1d*, *Galnt6*, *Rapgef5*, *Cdc42ep2*, *Kcna1*, *Anln*, *Galnt15*, *Pitx2*, *Cdh19*, *Trim66*, and *Col18a1*), 7 in F_1_ and F_3_ (*Ank1, Kiaa1024, Exph5, St8sia1, Kiaa1217, Ccdc109b*, and *Sptb*), and none of the same genes were differentially expressed in F_2_ and F_3_ or in all three generations (Fig. 7A). GSEA revealed enrichment of 47 gene sets in F_2_ (4 positive, 43 negative) and 41 gene sets in F_3_ (30 positive, 11 negative; Supplementary Data Table 1; NES > 1.6, FDR < 0.25). 5 gene sets in F_2_ and 6 in F_3_ were associated with known behavioural, metabolic, and molecular effects of sGC treatment (Table 1; NES > 1.6, FDR < 0.25), including feeding behaviour and immune function (Fig. 7B; NES > 1.6, FDR < 0.25). The custom HPA Activity gene set (Table 3) was not enriched in either F_2_ or F_3_ (Table 1; F_2_ NES = 1.52, FDR = 0.154; F_3_ NES = 1.06, FDR = 0.409); there was no effect on any individual classic HPA genes.

## Discussion

We demonstrate that prenatal treatment with sGC results in transmission of altered HPA response to stress, stress-associated behaviours, and gene expression to F_3_ via maternal and paternal lineages. These effects are sex- and age-dependent and diminish in advancing generations. The strongest HPA and behavioural effects of sGC treatment occur in female offspring from the paternal line, and F_1_ and F_2_ offspring are affected to a greater extent than F_3_ offspring. Molecular analyses suggest that the mPFC and PVN of female PT animals may be involved in the generation-specific effects of sGC treatment on behaviour. Transmission through the paternal line to the third generation of offspring strongly implicates epigenetic mechanisms in the male germ line as the mode for transgenerational transmission.

Consistent with the effects of sGC in children, we demonstrate an enhanced HPA response to stress in F_1_ juvenile female offspring^27^. Furthermore, we demonstrate maternal and paternal transmission of altered HPA activity to F_2_ and F_3_ offspring. While the HPA response to stress is reduced following maternal transmission^17,18^, we demonstrate paternal transmission of increased HPA response to stress to F_2_ females and F_3_ males. An interesting pattern emerges whereby exposure to sGC affects the stress response in F_2_ females and F_3_ males regardless of parental lineage. Sex- and generation-specific programming have been described following prenatal sGC^17^ and in other transgenerational studies^28^. However, such a complex pattern of effects is novel and suggests a role for sex chromosomes and/or imprinted genes. It is possible that sGC affect the expression of imprinted genes from the X-chromosome^29^ and that this contributes to parent-of-origin^30^ × offspring effects. In line with this hypothesis, it has been demonstrated that parentally imprinted genes may be involved in mediating the sex-specific effects of high fat diet (increased body size) in F_3_ female offspring following paternal transmission^21^.

Prenatal treatment with sGC programs hyperactivity in children and young animals^31,32^. We demonstrate hyperactivity in F_1_ females and males on PND 19 following antenatal exposure to sGC, and that this treatment results in increased locomotor behaviour in and open field in F_2_-F_3_ females on PND 19 following paternal transmission. While hyperactivity can be disruptive in modern society, there may be evolutionary advantages that contribute to the conservation and transmission of this phenotype (e.g. increased resource acquisition and parental attention^33^). This is the strongest and most consistent phenotype that we observe and it provides the first evidence for transgenerational transmission (i.e. paternal line to F_3_) following antenatal sGC treatment. Furthermore, these findings demonstrate that adaptive stress behaviours can be transmitted across generations.

It is important to note that variability in locomotor activity in the open field can impact the HPA response to stress of the open field. Therefore, since HPA activity was measured after the open field test, we cannot separate any effect that the animal’s activity while in the open field may have had on HPA response. While we observed increased locomotor activity in F_1_ and F_2_ PT females that coincided with an increased HPA response to stress for these animals, there was no concomitant increase in HPA activity for F_1_ males or F_3_ PT females. Furthermore, we have previously demonstrated that prenatal exposure to multiple courses of sGC result in increased locomotor activity in PND 10 female guinea pigs, but there was no effect on HPA response to stress^31,34^. Thus, while the relationship between stress-induced hyperactivity and increased cortisol production requires further investigation it is clear that prenatal exposure to sGC programs both the HPA and behavioural responses to stress, and that these effects manifest across multiple generations. Offspring were tested twice in the open field to determine the response to the combined stress of maternal separation and the open field (PND 19) and to the open-field alone (PND 24; post-weaning). While it is possible that habituation to repeated testing may have influenced the behavior of the animals in the open field on PND 24, any habituation would have occurred across both control and sGC treated animals, allowing for valid comparisons to be made between groups. Furthermore, the data from the PND 19 clearly demonstrate a robust phenotype of HPA and locomotor hyperactivity in response to stress in F_1_-F_3_ female offspring, PT. It is acknowledged that interactions may occur when repeated/multiple behavioural testing occurs in the same animal. However, this is an inherent limitation of this type of study; it is not ethically acceptable to produce a separate cohort for each individual test.

Treatment with sGC does not result in hyperactivity when F_1_ animals are in the home cage (non-stress environment). However, in F_1_ males, sGC treatment results in a phase advance of locomotor activity in anticipation of powerful entrainment signals (lights-on and feeding). Similar effects on circadian behaviour have been observed in rats that were exposed to prenatal stress and involve changes in the expression of clock genes^35,36^. While transgenerational transmission of altered circadian behaviour occurs in F_2_ and F_3_, the nature of the effect is different from earlier generations (i.e. change in amplitude of activity rather than a shift in the profile) and appears to diminish by F_3_.

Contrary to our hypothesis, treatment with sGC does not affect anxiety-like behaviour or attention in juvenile F_1_ offspring. It may be that testing in older animals^37^ or using alternative tests would reveal an effect of sGC in our model. Therefore, subsequent studies in older animals need to be undertaken. It is important to note that we assessed passive, automatic attentional processes (gating of irrelevant or unnecessary information), whereas other studies assessed active attention^38^. Exposure to sGC (F_0_) reduces anxiety-like behaviour in the open field for juvenile F_2_ females (PT) and F_3_ males (MT), and reduces attention in F_2_ females (PT). However, there is a contradictory trend towards increased anxiety-like behaviour in the acoustic startle chamber for F_3_ males (MT). Divergence in the response to the acoustic startle, and the open field have been reported in animals that display altered HPA activity^39^, and is likely due to the tests provoking different forms of anxiety-like behaviour (unavoidable and avoidable^40^).

Prenatal treatment with sGC reduces body temperature in F_1_ and F_2_ males (MT) and F_2_ females (PT). Similar effects of sGC treatment have been demonstrated in rats and involve impaired thermogenic signaling (thyroid hormone) in the PVN^41^. In the present study, the hypothermic effects of sGC transmit to the next generation through both parental lines. The fact that sGC reduce overall body temperature without affecting rhythmicity suggests that sGC may affect the magnitude of thermogenic signaling pathways (e.g. production of thyroid hormone) rather than circadian or pulsatile signaling.

Antenatal treatment with sGC does not appear to program an increase in HPA response to stress in F_1_ and F_2_ females by altering the expression of HPA regulatory genes in the mPFC or PVN. While this was somewhat surprising, it is important to highlight that the mPFC and PVN were isolated with the animals in a basal state, and future studies need to be undertaken to investigate HPA-related gene expression profiles in the stress-activated state. Alternatively, treatment with sGC may increase HPA responsiveness to stress by increasing the sensitivity of the PVN to stress signals. In support of this possibility, we identify a gene expression signature indicative of reduced inhibitory gamma-aminobutyric acid (GABA) signaling in the F_1_ PVN following treatment with sGC.

Molecular analyses in the mPFC reveal patterns of gene expression that correspond to the effects of sGC on stress-related behaviours. The increase in open field locomotor activity in F_1_-F_3_ female offspring (PT) is associated with reduced *Gnb1* expression (a signal transduction gene involved in regulating locomotor activity^42,43^) only in the F_1_ mPFC. Additionally, exposure to sGC reduces PPI only in F_2_ female offspring following paternal transmission, and this is associated with increased *Reln* (a cortical development gene involved in regulating attention^44^) expression in the mPFC of these animals. Clearly, further work is required to better define these associations.

GSEA of RNA-seq data in the PVN reveals patterns of gene expression associated with altered homeostasis in F_1_-F_3_ females (PT). While thermogenic signaling is reduced in F_1_ and F_2_, body temperature is reduced only in F_2_; it is likely that the depletion of this gene set in F_1_ has more to do with ATP synthesis than with thermogenesis. Indeed, many of the downregulated genes in the F_1_ and F_2_ PVN are integral components in the respiratory electron transport chain, including *Ndufs6* (encodes the first enzyme in the electron transport chain) and *Cyc1* (encodes part of the complex that transfers electrons to cytochrome c). These data indicate that prenatal exposure to sGC may also impact oxidative phosphorylation in the brain. This is an important finding that requires further investigation since oxidative phosphorylation is integral to the processing capabilities of the brain^45^. We also observe increased expression of circadian gene sets, including clock genes, in F_1_ females. These data support the hypothesis that antenatal sGC program circadian behaviour by altering the expression of regulatory clock genes (e.g. *Clock* and *Rora*). Maintenance of an intact blood-brain barrier (BBB) at the PVN is paramount to its ability to regulate homeostasis. GSEA reveals enrichment in F_1_ and F_3_ for gene networks that regulate BBB competency (e.g. collagen formation and extracellular matrix) following antenatal sGC treatment. Together with our previous study in the fetal guinea pig brain^46^, these findings demonstrate that prenatal sGC promote BBB competency and provide the first evidence that effects transmit to F_3_.

Treatment with sGC reduces DNA methylation and increases gene expression in the fetal brain^47,48^. We also demonstrate increased gene expression in the juvenile F_1_ PVN, but the effect diminishes in F_2_ and F_3_. GSEA reveals reduced expression of a one-carbon metabolism gene set in F_1_, which suggests that reduced DNA methylation may be involved in the upregulation of gene expression. Interestingly, exposure to sGC (F_0_) does not affect DNA methylation networks in the F_2_ or F_3_ PVN, and the mechanisms underlying the transcriptional effects of sGC in F_2_ and F_3_ remain to be determined.

We have also identified molecular signatures associated with risk factors for cardiometabolic disorders^2^ in the PVN of young F_1_-F_3_ female offspring, though we did not assess metabolic phenotypes in the present study. It is not surprising that weight gain or growth are not increased in these female animals since overweight and altered metabolic phenotypes emerge when animals are older or challenged with a high-energy diet^49,50^. However, we do observe increased growth in F_3_ male offspring following paternal transmission. These data suggest that sGC result in transgenerational priming of the central circuitry that promotes feeding behaviour and energy storage, this effect is present early in life, and may translate to altered body composition or growth in a sex-dependent manner.

Similar to other instances of transgenerational transmission, exposure to sGC results in complex patterns of effects that emerge and disappear as a function of age, sex, generation, and parental line of transmission^51^. While the programming effects of sGC generally decrease over time, paternal transmission results in more effects in F_2_ and F_3_ offspring when compared to maternal transmission; this implicates different mechanisms of transmission. The most likely mechanism governing maternal transmission is through altered maternal adaptation to pregnancy. Treatment with sGC has been shown to affect maternal HPA activity during the F_1_ pregnancy in mice^20^, and such alterations to the pregnancy environment create a ‘metabolic cascade’ that program the fetus to develop along a different trajectory. This cascade creates a self-perpetuating process that alters the next generation’s adaptations to pregnancy and propagates effects across multiple generations^52^. Since males were not involved in rearing offspring, the most likely mechanism governing paternal transmission to F_3_ is through the epigenome of sperm and/or seminal fluid. Recent transgenerational studies have provided strong support for the involvement of small non-coding RNAs in paternal transmission^53–55^. These RNA species are present in sperm/seminal fluid, can recapitulate the programmed phenotype when injected into an embryo, and are present at approximately twice the level on the X-chromosome as compared to autosomes.

## Conclusions

This study presents the first evidence that prenatal treatment with sGC results in transgenerational paternal transmission of hyperactivity and altered hypothalamic gene expression through three generations of young offspring. Female offspring appear to be more sensitive than male offspring to the programming effects of sGC, which suggests an interaction between sGC and sex hormones or sex-linked genes. Paternal transmission to F_3_ strongly implicates epigenetic mechanisms in the process of transmission, and small noncoding RNAs likely play a major role. The mechanisms by which sGC program similar phenotypes in F_1_-F_3_ differ between generations, but appear to involve altered transcription of genes in mPFC and PVN. Importantly, neither birth weight nor growth was reduced by sGC. Clearly prenatal treatment with sGC results in very long-term programming effects in the life of an individual, and effects reach into subsequent generations. The findings provide new perspectives on potential factors that may contribute to an individual’s vulnerability to disease development. Given the re-emergence of antenatal treatment with multiple courses of sGC, it is imperative that prospective and follow-up studies of human cohorts continue in order to determine the long-term effects of sGC in humans.

## Methods

### Animals and Treatments

Twelve week-old female Dunkin-Hartley guinea pigs (F_0_; Charles River; St Constant, QC, Canada) were singly housed within visual, olfactory and auditory contact of other animals. Food (Teklad Global Guinea Pig Diet # 2040; Envigo) and water were available *ad libitum*. Animals were maintained in temperature (23 °C) and humidity controlled rooms on a 12-hour light-dark cycle (lights on 0700 h, off 1900 h). F_0_ females were mated as previously described^56^. All protocols were approved by the Animal Care Committee at the University of Toronto in accordance with the Canadian Council on Animal Care.

Pregnant F_0_ females were injected with three courses of saline (Veh; 0.166 ml/kg; N = 40) or clinically relevant dose of sGC betamethasone (sGC; 1 mg/kg; Betaject phosphate-acetate mix - Sabex, Boucherville, QC, Canada; N = 40) on GD 40 & 41, 50 & 51, 60 & 61 as previously described^17^. Female and male F_1_-F_3_ offspring were randomly assigned (via dice roll) to juvenile or breeding streams. Investigator was not blinded to the group allocation. Offspring were weighed every 10 days from birth until postnatal day (PND) 40, and measures of body size were collected at birth and PND 20; food/water intake were not measured. Animals designated for breeding did not undergo any behavioural testing. Litters were weaned on PND 20 (water and Teklad Guinea Pit Diet were available *ad libitum*) and pair-housed in polycarbonate shoebox cages with an age-, sex-, and treatment-matched partner.

To investigate transgenerational maternal (MT) and paternal (PT) transmission, F_1_ and F_2_ female and male animals designated for breeding were mated with naïve animals (males and females, respectively, purchased from Charles River) to create MT and PT lines of F_2_ and F_3_ offspring (Fig. 1A and B). F_1_ and F_2_ pregnancies were undisturbed other than routine cage maintenance.

### HPA Testing

The HPA response to stress of open field (OF) exposure (PND 19 and 24; between 0800 h and 1000 h) was measured through salivary cortisol (animals freely chewed on cotton swabs). Samples were collected at 0 (pre-test in home cage), 30, 60, and 120 min. Saliva was stored at −20 °C until measurement of free cortisol by enzyme linked immunosorbent assay (Salimetrics LLC, State College, PA, USA) as previously described^57^. Analyses of cortisol for a given test (i.e. OF F_1_) were run in the same assay. Intra- and interassay coefficients of variance were 8.19% and 11.65%, respectively.

### Behavioral Testing

#### Open Field

Guinea pigs were tested for total locomotor activity, distance traveled, and percent of time spent in the outer zone of the open field (OF; Opto-max animal activity meter; Columbus Instruments, Columbus, OH, USA; clear Plexiglas box, 42.5 × 42.5 × 23 cm) as previously described^17,58^. Testing occurred for 30 minutes on PND 19 (prior to weaning) and PND 24 (after weaning) between 0800 h and 1000 h, and OF chamber was disinfected (Virox) between animals.

#### Acoustic Startle/Prepulse Inhibition

Offspring were tested for acoustic startle response (ASR; a measure of unavoidable anxiety-like behaviour) and prepulse inhibition of the startle response (PPI; sensory-motor gating, passive attention) in the SR-Lab Startle Response System (San Diego Instruments, San Diego, CA, USA; clear Plexiglas tube, 20 cm long × 8.89 cm diameter) as previously described^59^. Testing occurred for 20 min, including 5 min acclimatization, on PND 23 between 1400 h and 1600 h. Startle chamber was disinfected between sessions. Baseline ASR was determined by exposure to 4 pulses at 120 dB following acclimatization. Testing sessions consisted of the following 60 trials presented in a pseudo-randomized order as previously described^59^.

#### Activity/Body Temperature

Radiotelemeters (model TA-F40; Data Sciences International, St. Paul, MN, USA) were subcutaneously implanted on PND 33 to monitor locomotor activity and body temperature over a 24-hour period in the animal’s ‘home’ cage. Data were collected (uninterrupted) every 5 min beginning at 0900 h on PND 35, and summed into one-hour bins for analysis.

### Prefrontal Cortex and PVN Gene Expression: Quantitative Real-Time PCR

Juvenile guinea pigs were weighed and euthanized in the morning (0900–1100 h) by decapitation at PND 40 (43.6 ± 0.45). The brain was removed, weighed, dissected, and frozen on dry ice.

As strongest behavioural effects of prenatal sGC were observed in the F_1_-F_3_ paternal line females, this group underwent subsequent molecular analysis. The right frontal cortex and hypothalamus from F_1_-F_3_ paternal line females were cryosectioned at −20 °C. 1.0 mm diameter punches (Harvard Apparatus Inc., Holliston, MA, USA) of the mPFC cingulate cortex area 1 and infralimbic cortex (Fig. 6B), and the anteromedial hypothalamus, containing the entire PVN, (Fig. 7B) were collected. Other hypothalamic regions adjacent to the PVN may be represented in the anteromedial punches. RNA from punches was extracted using AllPrep Universal Kit (Qiagen). Bioanalyzer (RNA 6000 Pico LabChip, Applied Biosystems); determined all RNA samples had RIN ≥ 7. cDNA was made using SensiFAST cDNA synthesis kit (Bioline, London, England). qRT-PCR was run in triplicate (SensiFAST SYBER Hi-ROX 20 μl reaction, Bioline) and quantified by a CFX96 Real-Time System (Bio-Rad). Expression of target mRNA (Table 2) relative to *Gapdh* housekeeping gene was assessed using the 2^−ΔΔct^ method.

**Table 2:** qRT-PCR Primer Pairs.

	Forward	Reverse
*Mr*	AAACGTATCAAGCTCTACTTTAC	CCCATAGTGACATCCTGAG
*Gr*	TGTGAACTTTCCTGGCCGAT	GGTCCCGTTGTTGTTGAGGA
*Fkbp5*	CTGGCCATGTGCTACCTGAA	TCTGGCGGCTTTATTCTGGG
*Gnb1*	GCGAGCTTGACCAATTACGG	AGCCTGGAGTCTGTGAGAGAG
*Reln*	GCATTTACATCGGGCAGCAG	CTTCGGGGAGGCATTCAGTT
*Crh*	GCCGCACTGTCTCAGCTC	TTCACCTCTGCCTGCTATTTC
*Avp*	GACAACGCAGTGTCCTCTGT	AACAACCAGGCAACTTCCCA
*Sdcbp2*	ATGCGTCGTGCAGAGATCAA	CTGCACAAAGATCCCCTGGT
*Gabrd*	CCTACAGGTCGGTGGAGGTA	CGGGCGTAGATGTCAATGGT
*Acta2*	CGTGTGTGACAATGGTTCCG	TCAGGGTCAGGATTCCCCTT
*Gapdh*	TGTACTGGAGGTCAATGAAGG	GTCGGAGTGAACGGATTTG

Forward and reverse primer pairs used for qRT-PCR in the mPFC and PVN.

### PVN RNA-Sequencing

mRNA libraries were prepared using Illumina TruSeq V2 mRNA enrichment. Samples were sequenced (F_1_: Veh N = 5, sGC N = 5; F_2_: Veh N = 5, sGC N = 5; F_3_: Veh N = 6, sGC N = 6) on Illumina HiSeq. 2500 at 1 × 51 bp by the Donnelly Sequencing Centre. Reads (~45 million reads per sample) were aligned to the *Cavia porcellus* reference genome (>85% alignment, cavPor3.83) with Tuxedo Suit tools^60^ accessed through The Galaxy Project^61^. Subsequent analyses were performed in R (version 3.2.3). Gene read counts were determined with Genomic Alignments (version 1.6.3) as previously described^62^. Outliers were removed using Cook’s distance with default cut-offs^63^, and data were normalized by residuals with RUVSeq (version 1.4.0)^64^. Differential gene expression was assessed using EdgeR’s (version 3.12.1)^65,66^, general linear model likelihood ratio test, and FDR-corrected P < 0.05 was considered significant. Genotype permutations (1000) were computed in GSEA to determine gene set enrichment. Gene sets with FDR ≤ 0.25, P ≤ 0.01, and NES ≥ 1.6 met significance thresholds. Differential expression for 7 candidate genes assessed by qRT-PCR validated the RNA-seq data as there was a strong correlation between RNA-seq and qRT-PCR results (Supplementary Fig. 1; R^2^ = 0.9763; P = 2.69e^−05^).

Samples for F_3_ were sequenced in 2 batches. The first batch was composed of 6 Veh and 4 sGC. In the second batch, 2 Veh samples were re-sequenced with 2 additional sGC samples at 1 × 58 bp with sequencing depth of >65 million reads per sample and alignment of >85%. Batch effects were corrected using ComBat from R package sva (version 3.18.0)^67^ after which technical replicates were removed and data (Veh: N = 6, sGC: N = 6) were normalized by residuals with RUVSeq (version 1.4.0)^64^. Differential gene expression was assessed as described above.

### Data Availability

Data is available at Gene Expression Omnibus (GEO) repository, accession number GSE85822. Computer code is available upon request.

### Statistical Analyses

Analyses of all data (except RNA-Seq) were conducted using STATISTICA 12 (StatSoft, Inc., Tulsa, OK, USA). Investigator was blinded to the group allocation at the time of analysis, and un-blinded after completing analyses. All data are expressed as mean ± SEM; significance is defined as P < 0.05, trend is defined as 0.05 ≤ P < 0.075. Data from same-sex littermates were meaned to prevent litter bias. Sample sizes (N) correspond to independent litters, and not to the total number of offspring across all litters. Power analyses based on previous studies determined N ≥ 8 sufficient to account for inter-litter variability and detect effects in the tests performed. For RNA-sequencing, Scotty web tool^68^ determined that 4–6 samples per treatment (N = 4–6) with 40–60 million reads per sample allows for detection of 90–96% of genes with ≥1.5 fold change in expression with 80% power at P < 0.05. Following our hypotheses, separate analyses were conducted for sex (female and male), breeding line (MT and PT), and generation (F_1_, F_2_, and F_3_). Outliers were identified using the Rout method in Prism with the Q set to 1%^69^. Missing data points were imputed using the MD Imputation in STATISTICA 12 package^70^. Data from individual animals were excluded if no data were collected for a specific test or if >50% of data points were missing for a specific test. Cortisol area under the curve (AUC) was computed using the trapezoid rule with y = 0 as baseline for total AUC (AUC_T_) and y = the value at time 0 as the baseline for net AUC (negative and positive areas were summed; AUC_N_) as previously described^17^. Two-tailed student’s t-tests were used to analyze the total activity in the OF (Activity Units; AU), distance traveled during first 5 minutes in OF (cm), percent of time spent in outer zone during first 5 minutes in OF, AUC_T_ and AUC_N_ (ng/mL), acoustic startle response, 24-hour average body temperature (°C), organ:body-weight ratio, and fold-change in gene expression from Veh. Data sets with unequal variances between treatment groups were first log-transformed, and if the variances were still unequal a non-parametric Mann Whitney test was used. Two-way ANOVA with repeated measures and Tukey HSD post-hoc tests were used to analyze body measures (treatment × postnatal age; cm), weight gain (treatment × postnatal age; g), cortisol response to OF (treatment × time; ng/ml), PPI (treatment × prepulse intensity), and home-cage activity (treatment × time of day; AU).

## Electronic supplementary material

Supplementary Information

Supplementary Data Table 1
