# Supplementary material for: Prenatal Glucocorticoid Exposure Modifies Endocrine Function and Behaviour for 3 Generations Following Maternal and Paternal Transmission
Source: Sci Rep. 2017 Sep 18;7:11814. doi: 10.1038/s41598-017-11635-w (PMC5603559; doi:10.1038/s41598-017-11635-w)
Supplement: Supplementary file 1 — Supplementary Information [file 41598_2017_11635_MOESM1_ESM.doc]

PRENATAL GLUCOCORTICOID EXPOSURE MODIFIES ENDOCRINE FUNCTION AND BEHAVIOUR FOR 3 GENERATIONS FOLLOWING MATERNAL AND PATERNAL TRANSMISSION

Vasilis G. Moisiadis, Andrea Constantinof, Alisa Kostaki, Moshe Szyf, Stephen G. Matthews

**Supplementary Fig. 1. qRT-PCR validation of RNA-seq results. A)** Correlation of sequencing data to qPCR data (each point represents one gene): R^2^=0.9763; y=1.007x + 0.1897; P=2.69e-05. **B)** Comparison of fold change (from control) in expression observed from RNA-seq (closed bars) and qPCR (open bars).
